# Opportunities and Barriers in Telerehabilitation for Coronary Bypass Patients: A Case Study from the Faroe Islands

**DOI:** 10.1177/26924366251362187

**Published:** 2025-07-24

**Authors:** Bertil Christian Pløen Sivertsson, Alex Voss Gartner, Tetsuya Takahashi, Pernille Heyckendorff Secher

**Affiliations:** ^1^ Department of Health Science and Technology, Aalborg University, Aalborg, Denmark.; ^2^ Department of Physical Therapy, Faculty of Health Sciences, Juntendo University, Tokyo, Japan.; ^3^ Department of Nursing, University College of Northern Denmark, Aalborg, Denmark.

**Keywords:** cardiac rehabilitation, coronary artery bypass, heart diseases, patient compliance, telemedicine

## Abstract

**Introduction:**

Cardiovascular disease is a leading global cause of death, with coronary artery disease often requiring coronary artery bypass grafting (CABG). Inadequate rehabilitation increases health risks and costs, and low adherence to center-based rehabilitation has prompted interest in telerehabilitation. Despite technological advances, the global implementation of telerehabilitation for CABG patients remains underexplored. This study investigates factors influencing the implementation of a telerehabilitation device for CABG patients in the Faroe Islands.

**Materials and Methods:**

A qualitative case study design was used to identify factors influencing the b-near® system implementation at Suðuroy Hospital in the Faroe Islands. Data collection involved document materials, direct nonparticipant observations, and semi-structured interviews with seven participants. Analysis with NVivo followed principles by Kvale and Brinkmann and the Normalization Process Theory.

**Results:**

The case study identified both opportunities and barriers. At Suðuroy Hospital, the b-near system enhances training and communication between health care providers and patients, is easy for patients to manage, and eliminates the need for hospital transportation. However, barriers such as reduced social interaction and potential difficulties in understanding training audio were also revealed.

**Discussion:**

Several factors were identified, highlighting the importance of integrating these findings into Suðuroy Hospital’s implementation strategy. Overcoming barriers is crucial for the successful use of the b-near system and improved patient care. Further evaluation of the b-near system and similar devices in diverse health care settings is necessary to enhance patient outcomes and support broader implementation.

## Introduction 

Cardiovascular disease (CVD) is a leading global cause of death,^1^ with cases surging from 271 million in 1990^2^ to 607.64 million in 2020.^3^ During this period, CVD-related deaths increased from 12.1 million to 19.05 million. CVD includes heart and blood vessel conditions,^4^ with coronary artery disease (CAD) as a major contributor. CAD, characterized by plaque accumulation in the coronary arteries,^5^ accounts for one-third to one-half of all CVD cases globally, with an estimated prevalence of 200 million cases.^6,7^ The economic burden of CVD is substantial, with significant costs in the United States and Europe related to direct medical care, social services, and productivity losses.^8,9^ In Europe, CAD accounts for more than one-quarter of this financial burden.^9^

Management includes conservative medical therapy, percutaneous coronary intervention (PCI), and coronary artery bypass grafting (CABG).^10^ CABG is a significant surgical procedure designed to bypass atheromatous blockages in coronary arteries using harvested venous or arterial conduits.^11^ It remains a crucial intervention for patients with complex CAD, often offering superior survival outcomes compared with medical therapy or PCI alone in selected cases.^12,13^ The rise in global CVD prevalence^2,3^ and the reserved indication of CABG for advanced CAD,^5^ underscore the demand for this procedure. Risk assessment tools, such as the EuroSCORE and SYNTAX score, are often used to guide treatment decisions by evaluating preoperative conditions and anatomical complexity.^14^

CABG typically requires a hospital stay of nearly a week, and upon discharge, patients generally require a convalescence period lasting 2–6 weeks. During recovery, patients often face persistent issues such as heart failure, anemia, atrial fibrillation, pulmonary complications, and pain from thoracotomy and saphenectomy. Follow-up may reveal recurrent angina or acute coronary syndrome due to CAD progression or bypass failure, particularly with venous grafts, which are prone to stenosis.^15^ Patients are prescribed a complex medication regimen and advised to adopt a healthy lifestyle, including smoking cessation, a balanced diet, regular exercise, and stress management. Although adherence is initially high, it tends to decline over time.^16–18^

Cardiac rehabilitation (CR), comprising a comprehensive program that combines personalized and supervised exercise with education, plays a vital role in CABG care, facilitating recovery and long-term management of CAD.^15,19^ It is recommended for qualified CABG patients to promote physical and psychological well-being.^20–22^ Despite international guidelines and the documented benefits of CR,^23–28^ participation rates in rehabilitation programs for this patient group remain low.^29,30^ Challenges such as geographical distance between patients and CR facilities contribute to low participation in center-based cardiac rehabilitation (CBCR).^29,31,32^ Inadequate rehabilitation can lead to worse health outcomes and increased rehospitalization rates,^33–35^ impacting both patient well-being and health care costs.

Advancements in telehealth and remote monitoring have led to the development of home-based cardiac rehabilitation (HBCR) programs, designed to overcome barriers in traditional CBCR and improve accessibility, particularly for older individuals and those in rural areas.^36,37^ HBCR utilizes various telerehabilitation technologies, including wrist sensors,^38^ patient-centered web portals,^39^ smartphone apps,^40^ and real-time video-based technology,^41,42^ to offer a remote alternative to in-person care. This reduces the need for travel and benefits patients in remote locations,^43–48^ while improving access, patient engagement, and communication with health care providers.^49^ Although still evolving, European guidelines highlight its potential to improve participation and promote behavioral changes.^50^

Video-based telerehabilitation technologies are being evaluated within HBCR programs, with video-based communication emerging as a prominent tool for consultations between patients and health care providers.^49,51,52^ However, its implementation specifically for CABG patients remains underexplored.^53^ Addressing this gap is essential to ensure equitable access and improve outcomes.

This study aims to address this gap by exploring the factors influencing the implementation of the b-near® system, a Danish video call communication solution for patients with limited tech experience, in a CR program.^54,55^ The system, described by its developers as having an intuitive interface,^55^ has been translated into multiple languages, costs €745 and allows one-touch connection to health care providers. Its potential is particularly relevant for geographically challenging settings such as the Faroe Islands. A case study at Suðuroy Hospital will examine how b-near supports CABG rehabilitation, ensuring equitable access to advanced CR services.

## Materials and Methods

### Study design

A qualitative explorative case study design,^56^ incorporating principles from interpretive phenomenological and hermeneutic analysis, was used to explore the factors associated with the use of the b-near for CABG patients, focusing on its implementation at Suðuroy Hospital. This design aligns with the research objectives, enabling an in-depth exploration of real-world phenomena within their natural setting. The study was conducted in accordance with Consolidated Criteria for Reporting Qualitative Research guidelines.^57^

### Setting and participants

From February to March 2024, participants were recruited from the Physiotherapy Department at Suðuroy Hospital, one of the three main hospitals in the Faroe Islands,^58^ located in Tvøroyri on Suðuroy, the southernmost island. Faroese CABG patients face challenges when returning from Copenhagen University Hospital in Denmark to Suðuroy Hospital. The rugged terrain and unpredictable weather of the Faroese archipelago exacerbate transportation difficulties, hindering access to adequate rehabilitation services. Suðuroy Hospital serves the island’s population of 4,589^59^ and recorded 1,171 inpatients and 14,394 outpatients in 2023, offering 26 inpatient accommodations.^58^ The Physiotherapy Department had 8,352 patient contacts in 2023.

Participant selection aimed for data saturation, using a criterion-based purposeful sampling method.^56^ Eligible CABG patients received informational flyers and were invited to participate if they met the following criteria: (1) post-CABG, (2) enrolled in the CR program at Suðuroy Hospital, and (3) Danish or English-speaking. Informed consent was obtained in person, and signed forms were shared with researchers. Participants were enrolled in a 3-month CR program incorporating the b-near system. The program, managed by two physiotherapists, integrated either CBCR or b-near sessions into workflows. Eight participants were recruited, of whom one was excluded at the start of the CR program due to absence. There were no dropouts during the remainder of the CR period, leaving seven participants.

### Data collection

A triangulation approach was utilized, incorporating document materials, semistructured interviews, and nonparticipant observations. Document materials,^56^ including manuals, reports, and studies related to CABG and telerehabilitation, were reviewed throughout the study. This review was particularly intensive at the beginning to establish a foundational understanding before conducting interviews and observations. Interviews, guided by a Normalization Process Theory^60^ (NPT)-based guide, and observations, guided by a semistructured observation guide,^61^ were conducted 4–5 weeks after the CR program commenced to ensure adequate experience with the b-near system. Each interview lasted approximately 30 min, and observations of b-near training sessions, each lasting around 45 min, totaled about 5.5 h. Interviews were conducted either at Suðuroy Hospital or in the patients’ homes, while observations took place only in the patients’ homes. Both interviews and observations were conducted in person in Danish by team members (B.C.P.S. and A.V.G.). In several cases, participants’ wives were present during the interviews but remained passive. Interviews continued until thematic saturation was achieved. All interviews were audio-recorded and transcribed by B.C.P.S. and A.V.G., supplemented with field notes from observations made before each interview. Transcriptions were reviewed collaboratively to minimize individual biases. The recordings and field notes were securely stored on an encrypted USB device.

### Data analysis

Analysis of the empirical data was conducted using NVivo (version 12.0),^62^ which facilitated transcription, meaning coding, condensation, and interpretation of the data. The research team, comprising three males and one female, had diverse health care backgrounds (physiotherapist, occupational therapist, and nurse) and qualitative research experience, ensuring thorough analysis. The data were deidentified and interviews were transcribed verbatim, using a transcription key to handle filler words and pauses. A coding tree was developed, and primary coders (B.C.P.S. and A.V.G.) independently coded all transcripts based on Kvale and Brinkmann’s principles^63^ and the NPT framework, identifying key themes, subthemes, and quotations. Coding discrepancies were resolved through consensus, with P.H.S. reviewing and validating the coding applications, categories, and themes. After coding, participant statements and observations were condensed, summarizing lengthy statements and field notes into brief expressions to capture the main essence of the content. The interpretation process involved summarizing the analytical text within each respective code group. Drawing from Kvale and Brinkmann’s analysis-interpretation method,^63^ inspiration was taken to investigate the meaning of texts across three interpretation contexts: self-understanding, critical common sense understanding, and theoretical understanding. Participants reviewed the themes through member checks, confirming the accuracy of the representation without altering the findings or thematic groupings. ChatGPT (version 4.0)^64^ was used for grammar and language refinement. No content generation was performed by artificial intelligence.

## Results

In total, seven male CABG patients, aged 64–83 years (average 70.43 ± 6.08), were recruited. The majority (86%) were retired, and all had undergone CABG procedures at various times (Table 1 for participant demographics). The case study identified several opportunities and barriers related to the b-near system for CABG patients at the Physiotherapy Department of Suðuroy Hospital. Data from 105 pages of interview transcriptions and 30 pages of observation field notes were analyzed to extract key insights, which are organized into themes, subthemes, and representative quotations (Table 2 and Table 3).

**Table 1.** Participant Demographics

Participant ID	Gender	Age	Occupation	Year of CABG
P1	Male	83 years	Retired	2018
P2	Male	64 years	Shop assistant	2023
P3	Male	70 years	Retired	2016
P4	Male	69 years	Retired	2023
P6	Male	67 years	Retired	2022
P7	Male	72 years	Retired	2022
P8	Male	68 years	Retired	2020

CABG, coronary artery bypass grafting.

**Table 2.** Themes, Subthemes, and Quotes of Opportunities and Barriers from Interviews

Theme	Subtheme	Quotations
Opportunities:		
b-near facilitates training for CABG patients	Can be used for exercises	“*It’s still just those exercises, with elastic bands. It’s the one where you use both the legs and the arms and everything up and down.”* (P3)
	CABG patients benefit from b-near training	“*So*, *the training here really elevates the pulse, and I think that’s probably good for myself.”* (P6)
b-near facilitates understanding between health care provider and patient	Enables understandable communication between health care provider and patient	*“We find it very easy to talk to each other.”* (P8)
	Can be used for health care providers and patients to observe each other	*“She sees me, and I see her, and she calls. So, it couldn’t be better.”* (P3)
Patients can manage b-near without difficulty	The patient can use the b-near for telerehabilitation sessions	*“But that screen, there’s only one, it’s as simple as it can be. And it should be that way too.”* (P7)
	b-near becomes a part of the patients’ everyday life	*“So, it became a part of the family, that screen.”* (P8)
b-near eliminates the need for transportation to hospital	b-near overcomes rough weather transportation issues	*“So, if it’s stormy and the weather is bad. You can’t drive, so you just don’t drive.”* (P7)
	Less transportation is convenient for CABG patients	*“Yes, I think it’s fine since there are many who can’t get to the hospital. There are several who live on other islands that don’t have a hospital. It would be perfect.”* (P4)
Barriers:		
Lack of physical presence can be a barrier	Less social presence using b-near	*“It’s better to go out there, then there’s a bit of social interaction too. Then there is more. You move from machine to machine, I think that’s good.”* (P3)

CABG, coronary artery bypass grafting.

**Table 3.** Themes, Subthemes, and Quotes of Opportunities and Barriers from Observations

Theme	Subtheme	Quotations
Opportunities:		
b-near facilitates training for CABG patients	Can be used for exercises	*“The physiotherapist instructs him in high leg lifts and asks the patient to increase the pace, which he does.”* (P1)
	Can be used to challenge patients physically	*“The patient takes deep breaths. He becomes breathless and sweats on his forehead.”* (P3)
b-near facilitates understanding between health care providers and patient	Can be used for training instructions	*“The physiotherapist demonstrates the exercise over the screen with verbal instructions, and the patient performs the exercise accordingly.”* (P3).
	Enables understandable communication between health care provider and patient	*“Even though the patient is further away from the screen and is panting, the patient can still hear the physiotherapist, and vice versa.”* (P7)
	Can be used for health care providers and patients to observe each other	*“The patient can observe what exercises the physiotherapist is doing, which he then imitates himself. The physiotherapist can count repetitions through the screen as she can see the patient.”* (P4)
Patients can manage b-near without difficulty	The patient can use the b-near for telerehabilitation sessions	*“The patient presses the screen himself and receives the call. He steps back and waits for the physiotherapist.”* (P4)
Barriers:		
b-near training sound can hinder health care providers’ patient comprehension	Speaking simultaneously hinders comprehension for health care providers	*“The patient sometimes talks over the physiotherapist, and the physiotherapist has to ask the patient to repeat.”* (P4)
	Panting speech hinders health care providers from hearing the patient on screen	*“The physiotherapist asks the patient to repeat, but this is due to panting speech. When the speech is not panting, the physiotherapist responds understandably.”* (P7)

CABG, coronary artery bypass grafting.

### Opportunities of b-near implementation

Participants highlighted several benefits of using the b-near in their CR programs. The system facilitated training and exercises, with participants visibly challenged, as observed by sweating, panting, and breathlessness. They reported tangible benefits from the training, as illustrated by P6’s comment:

So, the training here really elevates the pulse, and I think that’s probably good for myself. (P6)

Suðuroy Hospital should document and analyze patient interviews and observations about the b-near’s role in facilitating training. This includes organizing qualitative feedback, tracking adherence, and assessing physical fitness changes. The hospital should form a multidisciplinary team to review these insights and consider individual patient perspectives for tailored interventions.

Participants valued the b-near’s role in enhancing communication between health care providers and patients. The b-near screen facilitated clear training instructions and allowed patients to observe and imitate health care providers’ exercises. As P4 noted:

The patient can observe what exercises the physiotherapist is doing, which he then imitates himself. The physiotherapist can count repetitions through the screen as he can see the patient. (P4)

Suðuroy Hospital should systematically document feedback on communication clarity, using the b-near for training, and analyze patterns. A multidisciplinary team should review these findings and evaluate how the b-near facilitates communication and collaboration. Individual patient experiences should also be considered for tailored support.

Ease of use was another positive aspect, with participants finding the b-near simple and manageable. Observations confirmed that all participants completed sessions without difficulty. P7 remarked:

But that screen, there’s only one, it’s as simple as it can be. And it should be that way too. (P7)

Suðuroy Hospital should document and assess feedback on the b-near’s ease of use, manageability, and integration into daily life. The hospital should then review how these factors impact patient engagement and satisfaction, using individual feedback to guide improvements.

Participants noted that the b-near removes the need for transportation, which is crucial in challenging Faroese weather. This is especially relevant for older individuals who may find driving unsafe in storms and for patients in remote areas without nearby hospitals. P7 highlighted this advantage:

So, if it’s stormy and the weather is bad. You can’t drive, so you just don’t drive. (P7)

Suðuroy Hospital should document and analyze how the b-near reduces transportation barriers. A multidisciplinary team should evaluate the impact on patient access and satisfaction and consider expanding telerehabilitation services and support resources based on patient feedback.

### Barriers of b-near implementation

Participants identified that the lack of physical presence when using the b-near could be a barrier, noting a reduction in social interaction compared with in-person training. P3 highlighted this issue:

It’s better to go out there, then there’s a bit of social interaction too. Then there is more. You move from machine to machine, I think that’s good. (P3)

Suðuroy Hospital should document and analyze feedback on how the lack of physical presence impacts social interaction and patient engagement. This involves assessing the reduction in social interaction, challenges compared with in-person sessions, and potential effects on motivation and adherence. Evaluating these aspects helps understand the implications for patient engagement in virtual training.

The b-near’s training sound sometimes hindered health care providers’ ability to comprehend patients, particularly due to simultaneous speaking and panting from physical exertion. This issue was observed with P4:

The patient sometimes talks over the physiotherapist, and the physiotherapist has to ask the patient to repeat. (P4)

Suðuroy Hospital should analyze how sound issues during b-near sessions affect comprehension. This involves examining how simultaneous speaking and panting interfere with communication and understanding. Insights from this analysis can inform how sound-related challenges impact the effectiveness of virtual training.

## Discussion

The results of this study highlight both opportunities and barriers for implementing the b-near system for CABG patients at Suðuroy Hospital. Applying the NPT framework, it is evident that current efforts primarily focus on reflexive monitoring, with participants actively assessing their experiences with the b-near and its implications for CABG care. Based on the analysis of themes and insights from interviews and observations, Suðuroy Hospital should consider adjusting its implementation strategy for the b-near system to better address the challenges faced by CABG patients, particularly given the unique characteristics of the Faroese environment that hinder access to adequate rehabilitation services. Despite these challenges, the Faroe Islands’ independence from EU medical device regulations^65^ could facilitate the use of the b-near system, potentially improving patient accessibility and outcomes. Moreover, the publicly funded health care system^66^ covers the costs of implementing the b-near system at Suðuroy Hospital in the Faroe Islands, alleviating financial barriers often found elsewhere and promoting equitable access to telerehabilitation services.

Participants agreed that the b-near facilitated their training, enabling them to perform exercises as instructed and feel physically challenged. This aligns with previous research showing similar improvements in exercise parameters for both HBCR and CBCR among patients with CVDs, including those undergoing CABG.^67–71^ Studies reported comparable enhancements in the incremental shuttle walk test and 6-min walk test between HBCR and CBCR participants.^68,72–74^ Furthermore, a randomized controlled trial by Maddison et al. indicated that telerehabilitation, particularly the REMOTE-CR program, is at least as effective as traditional CBCR in improving various cardiovascular health outcomes, including VO_2_max, physical activity levels, and overall health-related quality of life.^75^ The cost-effectiveness of the REMOTE-CR program compared with CBCR underscores its economic viability. However, unlike comprehensive telerehabilitation programs that integrate sensors for vital biomarker monitoring, b-near primarily uses video conferencing. Further research is needed to evaluate b-near’s specific efficacy for CR.

Effective communication is crucial for fostering patient motivation and engagement, both of which are essential for successful long-term rehabilitation outcomes.^37^ Patients are more likely to adhere to their rehabilitation programs when they feel understood and supported.^76^ Rathore et al. emphasize that health care providers play a key role in motivating and engaging patients, and the b-near system supports this by enabling clear and meaningful interactions.^37,77^ While the b-near was effective in facilitating communication between health care providers and patients, issues such as background noise during training sessions were noted, which could hinder understanding. To address this, guidelines should instruct patients using b-near to wait for the health care provider to finish speaking before responding and to refrain from replying if experiencing breathlessness.

Participants found the b-near easy to use, addressing some usability concerns identified in other studies.^52,75^ Unlike more complex systems that require multiple connected devices, the b-near offers a simpler alternative, particularly for elderly CABG patients. However, this ease of use must be balanced against the need for comprehensive biomarker data, such as heart activity from an electrocardiograph during rehabilitation.

The b-near’s introduction eliminated the need for hospital transportation, which is beneficial in the Faroese context, where weather conditions can pose significant challenges. By allowing patients to conduct rehabilitation sessions from home, the b-near enhances accessibility, a crucial factor in increasing participation rates in rehabilitation programs.^78^ Flexibility in scheduling is often cited as a significant advantage of HBCR over CBCR, leading to higher completion rates.^79^ However, one participant noted that the lack of in-person interaction reduced social engagement and accountability, highlighting a potential barrier to adherence.^78^ Remote monitoring, while motivational, does not fully replicate the benefits of real-time feedback from in-person sessions.^80^ Emotional distress, depression and anxiety can also hinder the effectiveness of HBCR, indicating the need for careful candidate selection.^81,82^ Therefore, while the b-near serves as a valuable assistive tool, it is not intended to replace in-person training but rather to support patients who face barriers to attendance.

A strength of this study is its inclusion of seven participants, which is appropriate for qualitative case studies seeking to provide in-depth insights into the phenomenon of interest. As Boddy^83^ notes, qualitative research emphasizes depth of understanding over breadth, and smaller sample sizes can still generate significant findings. In this study, data saturation was reached by the sixth interview, as no new themes emerged, indicating that the sample size was sufficient to capture the complexity of the subject matter. Another strength is the application of the NPT that provides a robust framework for analyzing the implementation of the b-near system. This theoretical grounding helps to clarify how factors influencing adoption can be systematically evaluated.

A limitation of this study is its reliance on a single case. Although this restricts direct generalizability,^56^ Flyvbjerg argues that well-selected single case studies, when conducted with depth and purpose, can provide valuable insights and significantly contribute to scientific knowledge.^84^ Another limitation is that all participants were male, which may limit the applicability to female CABG patients, who often present different clinical outcomes.^85^ While CABG is more common in men due to earlier and more severe CAD,^86^ research shows that women have poorer cardiac and cerebrovascular outcomes in the first five years postsurgery,^85^ including a higher risk of graft failure^87^ and perioperative myocardial infarction.^85^ These gender differences highlight the need for further research on outcomes in female patients to inform targeted clinical approaches.

## Conclusions

The b-near system shows promise in improving patient engagement and outcomes in CR programs, but several challenges must be resolved to maximize its effectiveness and user satisfaction. Future studies should assess the long-term efficacy of the b-near system and similar telerehabilitation devices across different health care settings, with a focus on their impact on patient outcomes and overcoming barriers to broader implementation.

## Data Availability

Data supporting the findings of this study are available upon reasonable request from the corresponding author.

## Abbreviations Used

CABG: Coronary artery bypass grafting
CAD: Coronary artery disease
CBCR: Center-based cardiac rehabilitation
CR: Cardiac rehabilitation
CVD: Cardiovascular disease
HBCR: Home-based cardiac rehabilitation
NPT: Normalization Process Theory
PCI: Percutaneous coronary intervention
VO_2_max: Maximal oxygen uptake
